# A feasibility study of the Mini-AFTER telephone intervention for the management of fear of recurrence in breast cancer survivors: a mixed-methods study protocol

**DOI:** 10.1186/s40814-017-0161-8

**Published:** 2017-07-20

**Authors:** Susanne Cruickshank, Emma Steel, Deborah Fenlon, Jo Armes, Karen Scanlon, Elspeth Banks, Gerald Humphris

**Affiliations:** 1 Faculty of Health Sciences and Sport, University of Stirling, Stirling, FK9 4LA UK; 2 College of Health and Human Sciences, Swansea University, Swansea, SA2 8PP UK; 3 Florence Nightingale Faculty of Nursing and Midwifery, King’s College London, London, SE1 8WA UK; 4 Breast Cancer Care, Central Office—Kennington Business Park Chester House, 1-3 Brixton Road, London, SW9 6DE UK; 5 National Cancer Research Institute, 407 St John Street, London, EC1V 4AD UK; 6 School of Medicine, University of St Andrews, St Andrews, KY16 9AJ UK

**Keywords:** Fear of cancer recurrence, Breast cancer, Breast care nurses, Mixed methods, Intervention

## Abstract

**Background:**

Fear of recurrence (FoR) is a major concern for patients following treatment for primary breast cancer, affecting 60–99% of breast cancer survivors. Mini-AFTER is a brief intervention developed to address this fear, that breast care nurses are ideally placed to deliver. However, their interest in delivering such an intervention is unknown and crucial to its introduction. This study aims to assess the perceived feasibility of the Mini-AFTER telephone intervention for implementation by breast care nurses to manage moderate levels of fear of recurrence among breast cancer survivors.

**Methods:**

A sequential explanatory mixed-methods design will be used, informed by normalisation process theory (NPT). The design will be guided by the stages of NPT. Specifically, understanding and evaluating the process (implementation) that would enable an intervention, such as the Mini-AFTER, not only to be operationalised and normalised into everyday work (embedded) but also sustained in practice (integration). Phase 1: all members on the UK Breast Cancer Care Nursing Network database (*n* = 905) will be emailed a link to a web-based survey, designed to investigate how breast cancer survivors’ FoR is identified and managed within current services and their willingness to deliver the Mini-AFTER. Phase 2: a purposive sample of respondents (*n* = 20) will be interviewed to build upon the responses in phase 1 and explore breast care nurses’ individual views on the importance of addressing fear of recurrence in their clinical consultations, interest in the Mini-AFTER intervention, the content, skills required and challenges to deliver the intervention.

**Discussion:**

This study will provide information about the willingness of breast care nurses (BCNs) to provide a structured intervention to manage fear of recurrence. It will identify barriers and facilitators for effective delivery and inform the future design of a larger trial of the Mini-AFTER intervention.

## Background

Despite continued reductions in breast cancer mortality, 60–99% of individuals treated for breast cancer live with an ongoing fear that the cancer will recur [1–5], and for which many seek help from health professionals. Fear of recurrence (FoR) is rated as one of the top-ranking concerns in breast cancer patients attending out-patient clinics with 62% wishing to discuss this issue with their clinician [6]. FoR may be exacerbated once treatment is complete and can significantly increase healthcare encounters as survivors seek reassurance in distinguishing between recurrence and treatment-related bodily changes [7]. FoR can be understood as a natural response to a cancer diagnosis, experienced on a continuum from none to very high. However, when severe, it can lead to distress, difficulty in coping and is a predictor of poorer physical health [8]. Prior work suggests health professionals lack skills and/or confidence to adequately recognise or address fears of breast cancer returning or progressing [1].

The Ottawa Fear of Recurrence Colloquium issued a Delphi study-derived definition of FoR as ‘fear, worry, or concern about the cancer returning or progressing’ ([9], p.3267). High levels of FoR are characterised by excessive checking, overvigilance, frequent intrusive thoughts, poor sleep and anxiety [10], and can compromise quality of life for several years following diagnosis and treatment [4, 11–13]. Individuals experiencing this level of FoR may require intensive specialist psychological support [14]. Humphris et al. developed the AFTER intervention for cancer patients with high levels of FoR [10, 15]. The AFTER intervention is based on Leventhal’s Self-Regulation Model which proposes that fears of illness are triggered by sensations which people interpret as symptoms [16, 17]. The acronym AFTER represents A = Assessment, F = Family, T = Thoughts and feelings, E = Examination and self-care and R = Returning of cancer and review. The fear of recurrence 4-item scale (FCR4) was used to determine the level of fear (score min 4 and max 20). Three items refer to different aspects of anxiety and worry about the cancer returning and the fourth item invites a rating of frequency of ‘waves of strong feelings about the cancer coming back’: a score of 10 on this scale approximates to the 60th percentile and is defined as moderate FoR; a score of 15 approximates to the 90th percentile and equates to a high level of FoR [10, 15]. The AFTER intervention is a manual-based psychological treatment and comprises six face-to-face sessions. Delivery is conducted by a suitably trained psychologist or specialist cancer nurse. It has been shown to benefit people with cancer such as head and neck [10, 18]. It is generally accepted that early assessment and brief structured intervention has the potential to prevent symptoms of FoR worsening and to improve QoL [19]. Evidence also suggests that cost savings could be made if high FoR is effectively treated, because patients with high FoR use more services or present later for tests to identify recurrence [19, 20]. Hence, this focus on intervening earlier, when FoR is at a moderate level, has led to the development of a less intensive version of the AFTER intervention, the Mini-AFTER intervention [21]. The reasoning for developing a brief version of the AFTER intervention was based upon the following premises. First, a preventive philosophy was considered beneficial, that is to intervene prior to FoR rising to a high level. Second, this preventative approach is strengthened by better understanding by health professionals of the impact of FoR on the individual with cancer when it reaches high levels. Third, BCNs are already working within all teams involved in breast cancer treatment and follow-up, making them well placed to deliver an intervention, and finally, the offer of a brief intervention fits into a stepped-care service approach which adopts a FoR trajectory model, i.e. not a simple one-off screen ‘test’ for FoR but rather a series of interventions. This approach is supported by recent evidence from the Netherlands on providing a stepped-care approach to FoR [22].

The Mini-AFTER intervention has been developed specifically to reduce moderate FoR in breast cancer survivors and is a single 30-min structured phone call designed to be made by BCN. It covers four key aspects associated with FoR: (1) ascertaining its significance and impact on cancer survivors’ lives; (2) the nature of the symptom(s); (3) triggers of FoR following treatment; (4) identifying potential confidantes among family or friends, and/or if there are difficulties discussing FoR. Fear is legitimised by acknowledging these responses and discussing ways they can be managed through relaxation procedures, normalising fears/beliefs, setting goals to reduce self-checking and seeking professional explanation and reassurance over unexplained symptoms. The intervention has also been shown to significantly reduce FoR [12, 23].

BCNs are highly skilled nurses who routinely provide emotional and psychological support to those with breast cancer [24] and were identified in the early development of the Mini-AFTER as being best placed to deliver the intervention [10]. Several studies have shown that nurse-led telephone interventions for breast cancer follow-up are feasible to deliver and they are associated with improved overall QoL. None, however, have specifically focused on FoR as a major concern [25–29]. The ability and confidence of BCNs to deal with this complex issue are key to successful delivery of the Mini-AFTER. However, it is unclear how ready and willing BCNs might be to deliver this kind of brief intervention. Given the emotionally laden nature of this particular problem and the concerns that health professionals have about addressing this need, it is not clear whether BCNs would be prepared to undertake this role, or what kind of training might enable them to feel confident to deal with any problems that may arise. There is a need, therefore, to understand if, and how, breast cancer patients’ fears are currently assessed and managed in practice, and what factors might contribute to the successful implementation of the Mini-AFTER intervention. This is a particularly important element of feasibility testing before moving to a pilot study of this intervention.

This protocol outlines a sequential explanatory mixed-methods study, designed to assess the acceptability and deliverability of a brief telephone intervention (Mini-AFTER) for the management of moderate fear of recurrence (FoR) in breast cancer survivors based on the views of BCNs. The findings from this study will inform the design of a larger trial of the Mini-AFTER intervention.

### Aims of study

The aim of this study is to determine the acceptability of BCNs implementing the Mini-AFTER intervention to identify and manage moderate levels of FoR among breast cancer survivors. The study objectives are:

1. To capture current approaches used by BCNs to identify and manage FoR
2. To understand the challenges and barriers for BCNs in assessing and managing FoR
3. To explore any BCNs perceived challenges and barriers to implementing the Mini-AFTER intervention
4. To understand what would enable BCNs to successfully implement the Mini-AFTER intervention in practice

## Methods

A mixed-methods sequential explanatory study has been designed [30], involving the collection of data in two consecutive phases (Table 1, adapted from Ivankova et al. [31]). In the first phase, using a survey, quantitative data will be collected and analysed to understand how BCNs identify and address their patients’ FoR currently and if there are any barriers to doing this in clinical practice (objectives 1 and 2). The researchers will review the results from phase 1 before proceeding to phase 2 and will build upon and elaborate further on areas of importance to the overall study. An example of how the researchers intend to do this will be to run an association (as part of the statistical analysis) between the scale values for the question ‘I always raise a discussion on FoR vs. I only discuss FoR if the patient raises it’ question with the question ‘Would you be interested in delivering the Mini-AFTER intervention in a future study?’ The interview schedule will include an area of questioning that can explore this further: i.e. explore BCNs’ individual views on the importance of addressing FoR in their clinical consultations, interest in the Mini-AFTER intervention, the content, skills required and challenges to deliver the intervention, ensuring objectives 3 and 4 are also fully addressed. Demographic data from the survey will guide the formation of the sample for the second phase of qualitative interviews.

**Table 1:** Flowchart of the mixed-methods sequential explanatory design

Phase	Procedure	Product
Phase 1: data collection	• Web-based survey	• Numerical data • Freetext responses
Phase 1: data analysis	• Multi-level models • Sensitivity analysis • Coding free text responses	• Descriptive statistics • Coded themes
Connecting Phase 1 and 2	• Purposive sampling frame determined by survey findings on specific domains • Develop interview questions to answer questions raised by survey findings	• Interview sample • Semi-structured interview schedule
Phase 2: data collection	• Individual semi-structured phone interviews (*n* = 20) • Verbatim transcription of interview audio recordings	• Interview transcripts
Phase 2: data analysis	• Framework analysis	• Thematic framework • Indexed and charted data • Mapping and interpretation of the data
Integration of Phase 1 and 2 results	• Interpretation of the survey data • Refinement to implementation process	• Discussion • Refinement of the Mini-AFTER intervention • Revision of Mini-AFTER study design • Feasible process for implementing Mini-AFTER in BCN practice • Future research

Adapted from Ivankova et al. [31]

Normalisation process theory (NPT) will be used in this study to provide a conceptual framework for this work [32]. NPT seeks to identify the component parts for understanding and evaluating the process (implementation) that would enable an intervention, such as the Mini-AFTER, not only to be operationalised and normalised into everyday work (embedded), but also sustained in practice (integration) [33]. This theoretical approach focuses on the work of the individual (in this study the BCN) and the group (supporting influence of the wider team) to enable an intervention to be normalised. NPT comprises of four main components (1) coherence (or sense-making); (2) cognitive participation (or engagement); (3) collective action (work done to enable the intervention to happen); and (4) reflexive monitoring (formal and informal appraisal of the benefits and costs of the intervention). The dynamic relationship between these components is influenced by the wider context of the intervention, e.g. organisational context, structures, social norms, group processes, barriers, challenges and conventions. Table 2 describes the four main NPT components in more detail, maps the type of questions/topic areas that will be explored against the framework and answer the objectives of the overall study. Examples of how these topics translated into the survey and/or interview questions are illustrated. These results will determine the next steps, and if a pilot RCT is indicated. If it is, it is likely to look at how study recruitment (of nurses and patients), training model, adherence to manual, patient response, and intraclass correlation (ICC) estimation would be achieved.

**Table 2:** Use of the normalisation process theory (NPT) topic guide for interview/survey questions

NPT component	Example question topics
Coherence	
Differentiation *How a set of practice differs from each other*	1. The relationship between knowing FoR is an area of concern and identifying how a new intervention aligns with everyday practice 2. The worth attributed to introducing a FoR intervention 3. Is the intervention easy to describe? 4. Is it different from other interventions? 5. Is there a shared sense of purpose to address FoR among women with breast cancer? 6. Who would the intervention benefit (thinking more broadly)? Interview: Whose responsibility is it to discuss FoR? Is there a shared sense of purpose to address FoR among breast cancer patients? How do you think the Mini-AFTER differs to your current method of assessing patients for FoR 7. Are the benefits likely to be valued by women with breast cancer? 8. Will an intervention fit with the overall goals and activity of the organisation where the BCN works? Interview: Can you envisage the Mini-AFTER changing your practice?
Communal specification *Working together to build a shared understanding of the aims, objectives and benefits of a way of working*	
Individual specification *Understanding specific tasks and responsibilities around a set of practice*	
Internationalisation *Understanding the value, benefit and importance of a set of practices*	
Cognitive participation	
Initiation *A core problem and whether or not key participants are driving it forward*	1. Are BCNs likely to think it is a good idea? 2. Can they see the point of the intervention easily? Interview: Would you be willing to invest time to attain competence? 3. What kind of skills do BCNs have when dealing with FoR now? Survey: See Table 2 ver2 4. Will they be prepared to invest time, energy and work in it? Survey: See Table 2 ver2 This includes: • Training – examples giving in the survey • Attaining competence to deliver intervention • Time to deliver intervention • Invest in changing practice environment
Enrolment *May need to organise or reorganise themselves – rethinking relationships*	
Legitimisation *The belief that an individual can make a contribution*	
Activation *Identify and define the actions and procedures required to sustain a new practice*	
Collective action	
Interactional workability * Work people do with each other and the other elements of their practices to enable them to operationalise it*	1. How will providing a specific intervention affect the work of the BCN? Interview: How would Mini-AFTER impact your workload Interview: Would Mini-AFTER promote or impede your work? 2. Do they think it would promote or impede their work? 3. What effect will it have on the support they offer to women? 4. Do they think it would change the patient/BCN relationship? 5. Is the work compatible with their existing work practices? Survey: Questions understanding current working practices 6. How would the addition of an intervention impact on their workload and that of their colleagues? 7. What impact will there be on resources such as time? 8. How does it fit with the overall goals of their organisation/wider policy agenda?
Relational integration *The knowledge that participants have to build accountability and mental confidence in the new intervention*	
Skill set workability *Allocation of work – who gets to do work in the trial*	
Contextual integration *Allocation of resources to ensure the intervention can be fully executed in practice*	
Reflexive monitoring	
Systematisation *Individuals may seek to determine how effective and useful it is for them and others*	1. Perceptions of benefit to patients or staff 2. What may be required to make the intervention workable in practice? 3. When would be an appropriate time to review the intervention? 4. Do they perceive issues associated with recruitment?
Communal appraisal *Participants together (formally and informally) to evaluate the worth of a set of practices – Is it working*?	
Individual approval *Work experientially as individuals, its effects on them and the context (an intervention that complicates and demands workload may well have low uptake even if beneficial to patient)*	
Reconfiguration *Modifying or redefining a practice to make it workable in practice*	

### Phase 1: web-based survey

Phase 1 will address objectives 1 and 2. Responses will be collected from BCNs via a web-based survey to investigate the following feasibility domains [34]:

- Acceptability: e.g. How helpful are formal assessment tools to assess patients for FoR?
- Demand: e.g. What is the estimated proportion of patients who experience moderate and high levels of FoR, and how is it currently managed in practice?
- Practicality: e.g. What support do BCNs currently receive to fulfil their role and help patients deal with psychological issues? What barriers do they encounter using assessment tools in clinical practice?
- Adaptation: e.g. Do BCNs receiving training to deal specifically with FoR?
- Integration: e.g. Would BCNs be interested in taking part in a training course for Mini-AFTER and what would this consist of?

The web-based survey will be conducted via the Bristol Online Survey tool [35], which is fully compliant with all UK data protection laws and is highly accessible across different organisations.

### Phase 1 sampling and recruitment

The survey link and participant information sheet will be sent via email to members enrolled on Breast Cancer Care’s Nursing Network database (*n* = 905). Breast Cancer Care is a UK-based breast cancer charity. This organisation hosts a database of 905 health professionals working directly with people affected by breast cancer, of which 65% are BCNs. A prerequisite of joining the network is that a health professional must spend at least 50% of their time working with people affected by breast cancer. This number is consistent with data published by Macmillan Cancer Support [36–39]. With no other national register of BCNs in the UK, this route is the most direct way for the research team to access the study population. However, further steps will be taken to increase awareness among BCNs across the UK about the study through hospital networks and regional BCN groups to enhance our overall recruitment.

Eligibility criteria (being a BCN) will be detailed in the email, participant information sheet and consent page. Six weeks after the initial email, a reminder will appear in an email bulletin, circulated by Breast Cancer Care. The survey will be open for a period of 12 weeks.

Demographic data will be gathered about the age of participants, number of years working with people affected with breast cancer, number of years qualified, job banding, clinical area they align with, i.e. surgery, oncology, medical, highest level of clinical attainment, number of BCNs in the respondents’ workplace, numbers of newly diagnosed patients with breast cancer in their hospital and respondents’ place of work.

Table 3 illustrates the flow of the topics through the survey and examples of questions from each topic section. The questions were developed by the team and guided by the NPT approach illustrated in Table 2. The survey will be piloted among the research team, nurses working on the helpline at Breast Cancer Care (*n* = 4) and BCNs working in the NHS (*n* = 2) to test access and responses before it is distributed. In order to reduce bias, these nurses will be excluded from completing the survey and future studies.

**Table 3:** Survey structure and example questions

Section	Example questions	Question type
1. Consent	• Do you consent to taking part in this survey?	• Multiple choice^a^
2. About you	• No. of years working with people affected by breast cancer? • How many breast care nurses work on your team? • Approx. what proportion of your role is spent in the following areas?	• Multiple choice • Multiple choice • Scale^b^
3. About the patients you meet with breast cancer	• What psychological care interventions do you provide to patients with breast cancer?	• Multiple choice
	*FoR definition provided*	
	• In your opinion, what proportion of your patients experience FoR at a moderate level? At a severe level?	• Multiple choice
4. About the work you do	• Approx. what proportion of patients do you discuss FoR with? • How is the issue of FoR generally raised? (I always raise a discussion on FoR vs. I only discuss FoR if the patient raises it) • In your experience, what factors trigger FoR in a breast cancer patient? • What barriers do you encounter using assessment tools in clinical practice?	• Multiple choice • Scale • Freetext^c^ • Freetext
4. About the support within your team	• What kind of formal support do you receive to fulfil your role? • How would you rate the support you get from your team when helping patients deal with psychological issues? (Extremely supportive vs. Not at all supportive) • How would you rate the formal support you receive to fulfil your role?	• Multiple answer^d^ • Scale • Scale
5. About your preferred support	• Have you received training to deal specifically with the area of FoR?	• Multiple choice
	*Brief explanation of the Mini-AFTER intervention provided*	
	• If you were offered a one-day training course to implement the Mini-AFTER intervention, would you accept it?	• Multiple choice
6. About other support services you use	• If you identify a patient with moderate FoR, which of the following services do you refer them to? With severe FoR? • How do you decide which service to refer your patient to when they have FoR?	• Multiple answer • Freetext
7. Follow-up	*Explanation of Phase 2 of the study provided*	
	• Would you be willing to take part in a telephone interview? • Would you be interested in delivering the Mini-AFTER intervention in a future study?	• Multiple choice • Multiple choice

^a^Multiple choice questions allow respondents to pick just one answer from a list of pre-defined answers

^b^Scale questions ask respondents whether they agree or disagree with a number of statements, to rate items on a scale, or to rank items in order of importance

^c^Freetext questions allow respondents to type their answers in their own words

^d^Multiple answer questions allow respondents to pick more than one answer from a list of pre-defined answers

This study has strong patient and public representation. Patient (EB) and Breast Cancer Care (KS) representatives are co-applicant and collaborator, respectively, and involved in all substantive study matters. EB has significant experience in patient involvement with research and her insight has complemented the clinical and research expertise of the research team in shaping the study design.

To develop an audit trail, a record of the data collection process will be kept throughout the study, including the selection of the purposive sample for Phase 2.

### Phase 2: qualitative interviews

Phase 2 will address objectives 3 and 4 but will also be grounded in the quantitative results from the first phase, allowing us to explore and elaborate further on areas of importance to the overall study.

### Phase 2 sampling and recruitment

The final survey question will invite participants to take part in a 40-min telephone interview. Depending on the number of participants agreeing to be interviewed, and following analysis of the data from phase 1, a purposive sampling matrix will be developed. If numbers allow, two demographic factors such as age, number of years qualified, years in current post or level of educational attainment will be combined with sub-groups based on the answers to the following two key questions which may predict willingness to use the Mini-AFTER intervention:

- How is the issue of FoR generally raised? (I always raise a discussion vs. I only discuss FoR if the patient raises it)
- How comfortable are you discussing FoR with your patients? (Extremely comfortable vs. Not at all comfortable)

To achieve the planned matrix and reach data saturation, an overall sample size of 20 will be required.

Prior to interview, an information pack about the Mini-AFTER intervention will be sent to each selected participant. This pack will be made up of a participant information sheet re-iterating the eligibility criteria, an online presentation and extracts from the Mini-AFTER manual. Interviews will be digitally audio recorded and conducted over the phone by ES.

Interviews will explore BCNs’ views on the importance of addressing FoR in their clinical consultations, and how the Mini-AFTER intervention could be successfully integrated in practice. Phase 2 will also provide valuable information regarding the design and perceived feasibility of a future RCT, including the following domains [40]:

- Eligibility—who (people affected by breast cancer) will participate in the trial?
- Recruitment—how will participants be recruited into the trial?
- Setting—where will the trial be conducted?
- Organisation—what training and resources will be needed to deliver Mini-AFTER?
- Delivery—how should Mini-AFTER be delivered?

Table 2 illustrates how the interviews questions will be guided by NPT and provides examples of questions for each NPT component.

### Validity of interview data

To ensure a consistent interview style is used with all interview participants, ES will be the sole interviewer. Direct anonymised quotations will be used to illustrate findings in any future publications, presentations or reports. Double coding and utilisation of NVivo’s coding comparison query [41] on 25% of the transcripts will ensure consistency and rigour across the whole of the data.

### Data analysis

The survey data will be collected, coded and entered into SPSS 22 [42]. Descriptive statistics will be used to summarise the sample characteristics and responses to questions. Multiple responses from the same centre will be controlled for using a clustering approach.

Descriptive analysis of cross-sectional data will be reported to understand how FoR is currently identified and addressed in breast cancer patients. Associations will be examined to investigate variation in these responses according to self-reported training level, experience and service environment. Barriers to formal assessment and systematic intervention of FoR in clinical practice will be investigated.

We will use conventional random intercept/slope regression models with continuous and binary-dependent variables using suitable procedures in STATA (xtmixed and xtmelogit). We will perform tests using these approaches to determine if a multi-level approach is required. That is, the two procedures xtmixed2 (for continuous response data) and xtmelogit2 (for categorical data response data) will provide a likelihood-ratio statistic to indicate if variance within clusters (i.e. the BCNs from the same cancer units) is significant. To estimate these multi-level models the ‘marginal likelihood’ has to be estimated using approximate methods [43]. This approach uses an adaptive quadrature procedure which enables efficient solutions to be obtained and reduced computation time. A sensitivity analysis will be performed to determine the appropriate number of integration points to obtain the most precise estimation.

Audio recordings from interviews will be transcribed verbatim, and all identifiable information will be removed from the transcripts. Framework analysis will guide the analysis of the qualitative data via a five-step process: (1) familiarisation with the data; (2) identifying a thematic framework based on emerging themes or issues in the data; (3) indexing the data in relation to specific themes; (4) charting the data based on the indexed themes; and (5) mapping and interpretation of the data to analyse the key characteristics in the charts (35). All members of the research team will be involved in at least one step in the process to ensure both researchers and lay representatives contribute their expertise at all stages of the study. However, ES, DF, SC and JA will be involved in all steps.

Coding of the data will be informed by the four main components of NPT (coherence, cognitive participation, collective action and reflexive monitoring, Table 2), but will also provide enough flexibility for any new themes that may emerge. Double coding will be employed on 25% of the transcripts to ensure any bias in the interpretation of the data is highlighted. Qualitative data will be coded and managed using NVivo 10 software [41].

Following analysis of the two sets of data, the research team will integrate the results to reflect the outcomes of the entire study. Firstly, the quantitative results will be interpreted to answer objectives 1 and 2. Secondly, the qualitative findings will enable the phase 1 data to be further clarified by explaining the statistical results and addressing detail for objectives 3 and 4. The final stage will be to discuss and group the findings from both sets of data to the NPT framework and feasibility domains to ensure the decision about how, or indeed, whether a larger trial of the Mini-AFTER intervention could be developed, ultimately leading to its implementation in practice, is achieved.

## Discussion

The prevalence of FoR and the major impact it has on breast cancer survivors’ quality of life, psychological state and use of healthcare services leads to the need for interventions to be developed to support breast cancer patients with both severe and moderate levels of FoR. There are a number of interventions being designed and tested [14, 15, 44, 45]; however, there is no routinely available intervention in practice to help support people with moderate FoR following treatment for cancer. Indeed there is evidence that some health professionals are not managing the issue of FoR even when it is brought to their attention [1]. This has the potential to harm breast cancer survivors as well as contribute to an increased use of healthcare services in an attempt to seek ways to cope with their fear [7, 46]. Delivery of a nurse-led telephone intervention to address FoR could not only relieve some of the pressure on healthcare services for follow-up cancer care, but also better serve the psychological needs of breast cancer survivors.

This study has been designed to provide an overview (Phase 1) as well as an in-depth insight (Phase 2) into how FoR is currently being managed in practice, and what factors will contribute to the successful implementation of the Mini-AFTER telephone intervention. Use of NPT will inform specific aspects of the implementation of this intervention, such as whether it would promote or impede BCNs’ work, what impact it would have on their resources and how it would fit with the overall goals of their organisation/wider policy agenda. Findings will also provide information to inform the development of an RCT to test the effectiveness of the Mini-AFTER intervention.

### Limitations

The study may be limited by low response rates. We are relying on BCNs opening their email from Breast Cancer Care and reading the contents. With the function of automatic sifting of email, we may find that some BCNs are not contacted through this route. The use of reminders may mitigate against this. Another limitation is that we are accessing BCNs through a single route rather than directly through their organisations. Although the Breast Cancer Care Nursing Network is the most comprehensive database of BCNs working in the UK, and the most comprehensive route to accessing BCNs across the country, the research team will raise awareness of the work among BCN through hospital and regional BCN networks.

## Abbreviations

BCN: Breast care nurse
FoR: Fear of recurrence
RCT: Randomised control trial
